# Monitoring Depth of Anesthesia Based on Hybrid Features and Recurrent Neural Network

**DOI:** 10.3389/fnins.2020.00026

**Published:** 2020-02-07

**Authors:** Ronglin Li, Qiang Wu, Ju Liu, Qi Wu, Chao Li, Qibin Zhao

**Affiliations:** ^1^School of Information Science and Engineering, Shandong University, Qingdao, China; ^2^Institute of Brain and Brain-Inspired Science, Shandong University, Jinan, China; ^3^Department of Anesthesiology, Qilu Hospital of Shandong University, Jinan, China; ^4^Tensor Learning Unit, RIKEN AIP, Tokyo, Japan; ^5^School of Automation, Guangdong University of Technology, Guangzhou, China

**Keywords:** autoencoder, LSTM, EEG, anesthesia, feature extraction

## Abstract

Electroencephalogram (EEG) signals contain valuable information about the different physiological states of the brain, with a variety of linear and nonlinear features that can be used to investigate brain activity. Monitoring the depth of anesthesia (DoA) with EEG is an ongoing challenge in anesthesia research. In this paper, we propose a novel method based on Long Short-Term Memory (LSTM) and a sparse denoising autoencoder (SDAE) to combine the hybrid features of EEG to monitor the DoA. The EEG signals were preprocessed using filtering, etc., and then more than ten features including sample entropy, permutation entropy, spectra, and alpha-ratio were extracted from the EEG signal. We then integrated the optional features such as permutation entropy and alpha-ratio to extract the essential structure and learn the most efficient temporal model for monitoring the DoA. Compared with using a single feature, the proposed model could accurately estimate the depth of anesthesia with higher prediction probability (*P*_*k*_). Experimental results evaluated on the datasets demonstrated that our proposed method provided better performance than the methods using permutation entropy, alpha-ratio, LSTM, and other traditional indices.

## 1. Introduction

Electroencephalogram (EEG) signals have been widely used in various clinical applications including disease diagnosis and monitoring the depth of anesthesia (Zhang et al., 2001; Bruhn et al., 2006; Jameson and Sloan, 2006). Usually, the anesthesiologist doctors ask the patients some questions to monitor and estimate the DOA. This is inaccurate in clinical practice, and the accuracy of anesthesia monitoring depends on the experience of anesthesiologists. During the operation, misjudgment of the DoA of patients is serious and dangerous. If DoA is not deep enough, the patient may be awake during the operation and suffer from great psychological trauma. However, if too much anesthetic is used, the patient will be in deep anesthesia, which is not conducive to the patient's recovery and can even be life-threatening. Therefore, it is important to monitor the DoA exactly. Hence, EEG-based methods have been adopted as efficient clinical monitoring techniques due to the temporally varying and convenient features.

In recent years, many methods have been developed for monitoring the DoA index (Jiao et al., 2018; Jin et al., 2018). The bispectral (BIS) index was proposed by Rampil (1998), defining the DoA index in a range of 0–100. This algorithm has certain limitations in the burst suppression pattern (BSP), giving a high BIS index, while the patient is still in the state of anesthesia (Kearse et al., 1994). The EEG signal is non-stationary and exhibits non-linear or chaotic behaviors (Elbert et al., 1994; Natarajan et al., 2004). Many studies have shown that nonlinear analysis can be used for EEG in medical applications. Therefore, feature extraction of EEG signals based on nonlinear dynamics is widely used in the monitoring of anesthesia depth, for example, the Hurst exponent (Alvarez-Ramirez et al., 2008), detrended fluctuation analysis (Jospin et al., 2007), entropies (Bruhn et al., 2000; Chen et al., 2007), and a frequency band power ratio (Drummond et al., 1991).

Wavelet transform is an effective tool for extracting and analyzing the essential structure of signal in the time-frequency domain (Rezek and Roberts, 1998). As a method for identifying the time-frequency spectrum, wavelet transform can automatically adjust the size of the time window and better match the frequency characteristics of the signal; it is an ideal tool for signal analysis and processing. Therefore, many researchers had developed various wavelet entropy algorithms for DoA monitoring based on the wavelet transform, such as Shannon Wavelet entropy (SWE), Tsallis wavelet entropy (TWE), and Renyi wavelet entropy (RWE) (Rosso et al., 2006; Särkelä et al., 2007; Maszczyk and Duch, 2008). Wavelet entropy can represent the relative energy associated with the frequency band and detect similarities between signal segments (Puthankattil and Joseph, 2012; Benzy and Jasmin, 2015). It can measure the degree of signal order/disorder. Permutation entropy is a complexity measure for time series analysis. It is simple and has low computational complexity, which makes it useful for monitoring dynamic changes in complex time series. It is robust against artifacts in EEG in the awake state (Shalbaf et al., 2013). However, due to its high-frequency waves during the suppression period, permutation entropy is not effective in deep anesthesia (Cao et al., 2004; Li et al., 2008, 2010; Olofsen et al., 2008). Sample entropy was developed based on approximate entropy, which estimates irregularities and complexity by reconstruction of time series. Compared to the approximate entropy, the sample entropy eliminates self-matching, has less dependence on the length of the time series, and is more consistent when compared over a wide range of conditions (Richman and Moorman, 2000; Yoo et al., 2012). It can track the state of brain activity under high doses of anesthetic drugs but requires noise-free data (Shalbaf et al., 2012). Several different ratios of electrical activity in various frequency bands have been proposed as indices of anesthesia depth in previous studies (Drummond et al., 1991). Shah proved that the ratio of alpha and beta frequency to delta frequency power appears to be a useful tool for identifying stages of isoflurane anesthesia (Shah et al., 1988).

As well as traditional signal processing methods, learning-based methods have been widely used in EEG signal processing and have achieved good results (Zhang et al., 2015, 2018; Wu et al., 2019). In recent years, learning-based methods have also achieved good performance for monitoring DoA. As stated in Shalbaf et al. (2013), an artificial neural network was used to classify the DoA index with extracted feature sample entropy and permutation entropy. The various features extracted from the EEG represented different aspects of the EEG, so using multiple parameters to assess the depth of anesthesia was effective. Saffar also integrated the Beta index, SWE, sample entropy, and detrended fluctuation analysis as multiple features, and an adaptive neuro-fuzzy inference system was used for classifying the stages of DoA (Shalbaf et al., 2018). By applying the five indices of middle frequency, spectral edge frequency, approximate entropy, sample entropy, and permutation entropy as the inputs of the artificial neural network, Liu obtained the combination index and found that the combination of these variables was more accurate than a single index for monitoring DoA (Liu et al., 2016). Liu et al. (2019) extracted the EEG spectrum information as the input of a CNN and trained the CNN to classify the DoA, which achieved better results.

A Recurrent Neural Network (RNN) is a special neural network with a memory function, and it can effectively use temporal information to analyze time series. However, the traditional RNN model has the problem of gradient disappearance or gradient explosion, the long short-term memory network (LSTM), an improvement of RNN, solves this problem to a certain extent (Hochreiter and Schmidhuber, 1997). LSTM has been successfully applied in various fields such as handwriting recognition (Graves and Schmidhuber, 2009), machine translation (Sutskever et al., 2014), speech recognition (Graves et al., 2013), and so on.

An autoencoder can learn a representation of the input data efficiently through unsupervised learning (Vincent et al., 2008; Baldi, 2012). Li et al. (2015) used the Lomb-Scargle periodogram and a denoising autoencoder to estimate the spectral power from incomplete EEG. The results showed that this method is suitable for decoding incomplete EEG. It has been proved that a denoising sparse autoencoder can extract the features of data and improve the robustness of those features (Meng et al., 2017). Qiu et al. (2018) proposed a novel method of seizure detection based on a denoising sparse autoencoder, which achieved high classification accuracy in seizure detection.

In this paper, an anesthesia depth monitoring method based on a sparse denoising autoencoder (SDAE) and LSTM is investigated with a combination of hybrid features. We preprocessed an EEG signal containing noise through a sixth-order Butterworth filter. The permutation entropy, sample entropy, wavelet entropy, frequency band power, and frequency spectrum were then extracted as features and input into the SDAE-LSTM (Sparse Denoising Autoencoder and Long Short-Term Memory) network to estimate the DoA. The combination of these features compensates for the shortcomings of individual features and is the optimal combination, having been proven to be effective. An SDAE combined with LSTM can take advantage of the temporal information in EEG, increase the robustness of the system, and remove noise-containing information. The final experimental results demonstrated that our proposed hybrid method can achieve higher prediction probability (*P*_*k*_) and provide better prediction performance than the traditional approaches.

The remainder of this paper is organized as follows. In section 2, we introduce the dataset and the baseline methods commonly used for DoA monitoring and our designed SDAE-LSTM network. Section 3 presents the experimental results of the SDAE-LSTM performance and compare it with other methods. Finally, Sections 4 and 5 provide discussion and conclusions.

## 2. Materials and Methods

### 2.1. Data

The dataset used for evaluation was from a previous study (McKay et al., 2006) and collected at the Waikato Hospital in Hamilton, New Zealand, which contains 20 patients aged 18–63 years old. These patients were scheduled for elective general, orthopedic, or gynecological surgery. The experiment was reviewed and approved by Waikato Hospital ethics committee and all subjects provided their written informed consent (McKay et al., 2006).

All trials were performed under the conditions specified by the American Society of Anesthesiologists. The raw EEG, unprocessed sevoflurane concentration, processed end-tidal sevoflurane concentration, RE, and SE of each patient were recorded in detail in the dataset. The commercial GE electrode system, which consisted of a self-adhering flexible band holding three electrodes, was applied to the forehead of each patient to record EEG data (100/s). The end-tidal sevoflurane concentration recorded from the mouth was sampled at 100/s (McKay et al., 2006). A plug-in M-Entropy module was used to measure the response entropy (RE) (0.2/s) and state entropy (SE) (0.2/s); its sampling rate is 1,600 Hz, the frequency bandwidth is 0.5–118 Hz, and the amplifier noise level is <0.5uV. Patients first inhaled fresh gas at 4L/min and where then given 3% sevoflurane for 2 min, followed immediately by a 7% inspired concentration. When RE had decreased to 20, 7% sevoflurane was continued for a further 2 min. Finally, sevoflurane was turned off.

The dataset we used records the raw EEG, unprocessed sevoflurane concentration, processed end-tidal sevoflurane concentration, RE, and SE of each patient. We did not segment the EEG signal and used a continuous signal throughout the whole process.

### 2.2. Feature Extraction

#### 2.2.1. Sample Entropy

Sample entropy (SampEn) was developed by Richman and Moorman (2000) to represent the complexity of finite time series. Larger values of SampEn reflect a more irregular signal. Given a time series *x*(*i*), 1 ≤ *i* ≤ *N*, it can be reconstituted as *N* − *m* + 1 vectors *X*_*m*_ (*i*), defined as:

$$X_{m}\left(i\right)=\left\{x\left(i\right),x\left(i+1\right),\ldots ,x\left(i+m-1\right)\right\},i=1,2,\ldots ,N-m$$

Let *d* be the distance between the vectors *X*_*m*_ (*i*) and *X*_*m*_ (*j*), which is given by:

$$\begin{array}{r} d_{ij}^{m}=d\left[X_{i}^{m},X_{j}^{m}\right]=\max\left(\left|x\left(i+k\right)-x\left(j+k\right)\right|\right),\\ \;k=0,1,\ldots ,m-1 \end{array}$$

$C_{i}^{m}\left(r\right)$ is the probability that *X*_*m*_ (*j*) is within distance *r* of *X*_*m*_ (*i*), calculated as:

$$C_{i}^{m}\left(r\right)=\frac{n_{i}\left(m,r\right)}{N-m+1},i=1,N-m$$

where *n*_*i*_ (*m, r*) is the number of vectors *X*_*j*_ that were similar to *X*_*i*_ subject to *d*(*X*_*i*_, *X*_*j*_) ≤ *r*. When the embedding dimension equals *m*, the total number of template matches is:

$$A\left(m,r\right)=\frac{\sum_{i=1}^{N-m}C_{i}^{m}\left(r\right)}{N-m}$$

Setting *m* = *m* + 1 and repeating the above steps, the SampEn of the time series is estimated by:

$$\text{SampEn}\left(r,m,N\right)=-\text{Ln}\frac{A\left(m+1,r\right)}{A\left(m,r\right)}$$

where Ln is the natural logarithm. The SampEn index is influenced by three parameters *N*, *r*, and *m*. *N* is the length of the time series, *r* is the threshold that determines the similarity of the patterns, and *m* is the length of the compared sequences. In this paper, we set *N* = 500, *r* = 0.2, and *m* = 2. The parameters are selected according to Bruhn et al. (2000) and Liang et al. (2015).

#### 2.2.2. Permutation Entropy

Permutation entropy (PeEn) provides a simple and robust DoA estimation method with low computational complexity. It quantifies the amount of regularity in the EEG signal, and takes the temporal order of the values into account (Li et al., 2008). Given a time series *X*_*N*_ = [*x*_1_, *x*_2_,… , *x*_*N*_] with *N* points, *X*_*N*_ can be reconstructed as:

$$\begin{array}{r} X_{i}=\left\{x\left(i\right),x\left(i+\tau \right),\ldots ,x\left(i+\left(m-1\right)\tau \right)\right\},\\ \;\text{i}=1,2,\ldots ,N-\left(m-1\right)\tau \end{array}$$

where τ is the time delay, and *m* denotes the embedding dimension. Then, *X*_*i*_ can be rearranged in an increasing order:

$$\begin{array}{l} \{x\left(i+\left(j_{1}-1\right)\tau \right)\leq x\left(i+\left(j_{2}-1\right)\tau \right)\leq \ldots \\ \;\leq x\left(i+\left(j_{m}-1\right)\tau \right)\} \end{array}$$

There are *J* = *m*! permutations for *m* dimensions. The vectors *X*_*i*_ can be represented by a symbol sequence in which each permutation is considered a symbol. For the time series *X*_*N*_, the probabilities of the dissimilar symbols for the time series *X*_*N*_ are named *P*_1_,… , *P*_*j*_. Based on Shannon entropy, permutation entropy can be defined as:

$$PE=\frac{\sum_{j=1}^{J}P_{j}\text{Ln}P_{j}}{\text{Ln}J}$$

The calculation of PermEn is dependent on the length of the time series *N*, the length of the pattern *m*, and the time lag τ, which are *N* = 500, *m* = 4, and τ = 1. The parameters are selected as proposed in Su et al. (2016).

#### 2.2.3. Wavelet Entropy

Wavelet entropy is based on wavelet transform with multiple scales and orientations (Särkelä et al., 2007). A suitable wavelet base is selected, and the original signal is developed at different scales, where *C*_*j*_ (*k*) is the decomposition coefficients at each scale *j*. The wavelet energy *E*_*j*_ of a signal is defined as follows:

$$E_{j}=\overset{L_{j}}{\underset{k=1}{\sum }}|C_{j}\left(k\right){|}^{2}$$

where *L*_*j*_ denotes the number of coefficients at each decomposition scale. Therefore, the total energy of the signal can be expressed as:

$$E_{total}=\underset{j}{\sum }E_{j}=\underset{j}{\sum }\overset{L_{j}}{\underset{k=1}{\sum }}|C_{j}\left(k\right){|}^{2}$$

Then, wavelet energy is divided by total energy to obtain the relative wavelet energy at each scale *j*:

$$p_{j}=\frac{E_{j}}{E_{total}}=\frac{\sum_{k=1}^{L_{j}}{\left|C_{j}\left(k\right)\right|}^{2}}{\sum_{j}\sum_{k=1}^{L_{j}}{\left|C_{j}\left(k\right)\right|}^{2}}$$

Finally, the wavelet entropy is calculated by:

$$S=-\underset{j}{\sum }p_{j}\text{log}\left(p_{j}\right)$$

#### 2.2.4. Alpha-Ratio

The alpha-ratio is the logarithmic relative power of two distinct frequency bands and can be calculated as follows:

$$\;alpha-ratio=\text{log}\frac{E_{30-42.5Hz}}{E_{6-12Hz}}$$

where *E*_30−42.5_ and *E*_6−12_ represent spectral energy in the 30–42.5 and 6–12 Hz bands, respectively (Drummond et al., 1991; Jensen et al., 2006).

### 2.3. Our Work

#### 2.3.1. Long Short-Term Memory (LSTM)

**Figure 1:**
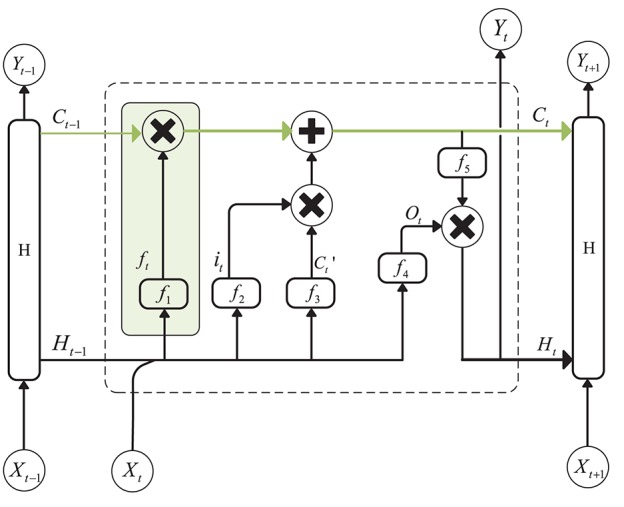
Structure of Long Short-Term Memory. The green line above the graph represents the cell state. The green box represents the gate, which controls the updating of the cell state.

A recurrent Neural Network is an efficient tool for sequential data analysis, such as EEG signal processing. The emergence of LSTM, as an improvement of the RNN network model, plays a significant role in solving the problem of gradient disappearance during RNN training. LSTM is a kind of special RNN model, and, in order to make the gradient flow for long durations, LSTM introduces self-loops and propose the concept of a gate. Compared with an ordinary recurrent network, each cell has the same inputs and outputs but more parameters (Goodfellow et al., 2016). The structure is shown in Figure 1 (Hochreiter and Schmidhuber, 1997):

The cell state update formula is as follows:

$$C_{t}=f_{t}C_{t-1}+i_{t}{C_{t}}^{\prime }$$

${C_{t}}^{\prime }$ is the candidate values created by a *tanh* layer:

$${C_{t}}^{\prime }=\tanh\left(W_{xc}X_{t}+W_{hc}H_{t-1}+b_{c}\right)$$

In the above equation, *W*_*xc*_ and *W*_*hc*_ are the weights of the input layer *X*_*t*_ at the current moment and the hidden layer *H*_*t*−1_ at the previous moment. *b*_*c*_ is the bias, and *f*_*t*_ and *i*_*t*_ are the forgetting gate unit and the input gate unit, respectively. The formulas for these two parameters are as follows:

$$f_{t}=f_{1}\left(W_{xf}X_{t}+W_{hf}H_{t-1}+b_{f}\right)$$

$$i_{t}=f_{2}\left(W_{xi}X_{t}+W_{hi}H_{t-1}+b_{i}\right)$$

where *f*_1_ and *f*_2_ are sigmoid functions that can map the value of the control coefficient between 0 and 1. And, the hidden layer *H*_*t*_ at the current moment can be written as:

$$H_{t}=o_{t}*f_{5}\left(C_{t}\right)$$

*o*_*t*_ is the output gate unit, and its expression is:

$$o_{t}=f_{4}\left(W_{xo}X_{t}+W_{ho}H_{t-1}+b_{o}\right)$$

*f*_4_ is also a sigmoid function. *W*_*xo*_ and *W*_*ho*_ represent the weights of the input layer *X*_*t*_ and the hidden layer *H*_*t*−1_ to the output gate, and *b*_*o*_ is the bias of the network. *f*_5_ is an activation function, such as *tanh*.

The LSTM must be trained to regulate the weights and biases. One of the most commonly used training algorithms is Bayesian regularization back-propagation.

#### 2.3.2. Autoencoder

**Figure 2:**
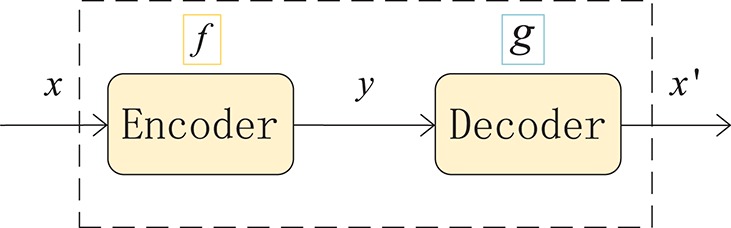
Structure of an autoencoder. The network consists of two parts: an encoder represented by the function *f* and a decoder, *g*. The encoder compresses the input into a hidden layer representation, and the decoder reconstructs the input from the hidden layer.

An autoencoder (AE) can be understood as a system that attempts to restore its original input, as shown in Figure 2, and it is a kind of neural network. The dotted blue box is an AE model that consists of two parts, an encoder and a decoder. The encoder converts the input signal *x* into a hidden representation *y*, and the decoder recovers *y* into an output signal *x*′, which is a reconstructed *x*.

y=f(x)

$$x^{\prime }=g\left(y\right)=g\left(f\left(x\right)\right)$$

The purpose of an autoencoder is to recover the input *x* as much as possible. In fact, we usually focus on the encoding of the middle layer, or the mapping from input to encoding. In other words, in the case where we force the encoding *y* and the input *x* to be different, the system can also restore the original signal *x*, and then the encoding *y* already carries all the information of the original data, which is a effective representation of the automatic learning of the original data.

**Figure 3:**
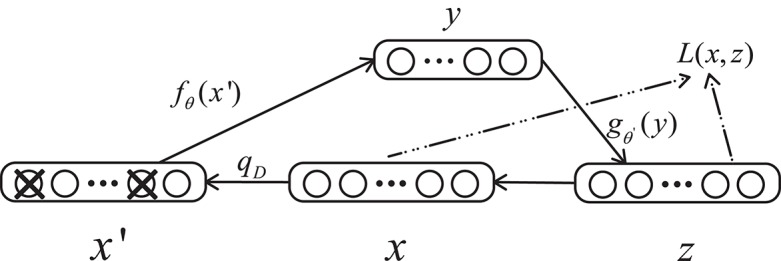
Structure of a denoising autoencoder. *x* represents the input data, *x*′ is the corrupted input, and *L* is the loss function.

A denoising autoencoder (DAE) is an extension of an autoencoder and was proposed by Vincent et al. (2008). To prevent over-fitting problems, noise is added to the input data (the input layer of the network), which makes the learned encoder *W* more robust and enhances the generalization ability of the model. A schematic diagram of a denoising autoencoder is shown in Figure 3. In Figure 3, *x* is the original input data, and DAE sets the value of the input layer node to 0 with a certain probability to get the input *x* containing noise (Vincent et al., 2010).

#### 2.3.3. Our Proposed Method:SDAE-LSTM

**Figure 4:**
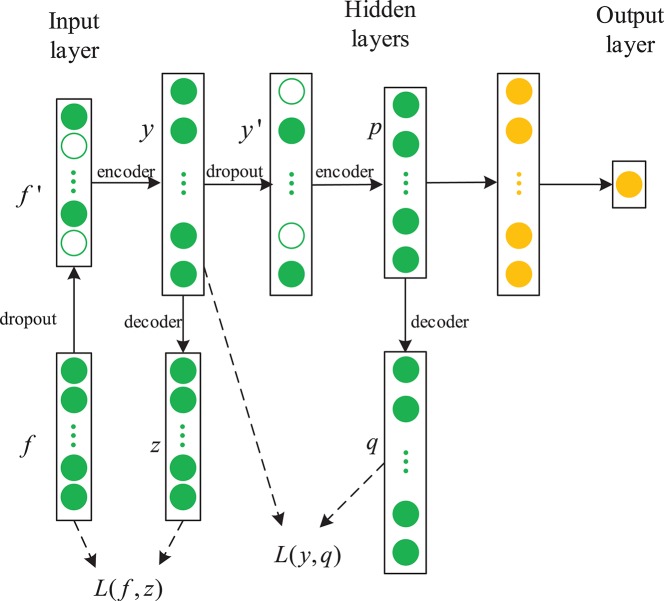
Structure of an SDAE-LSTM network. The green circles represent the layers of SDAEs and the yellow circles are LSTM layers. *f*′ represents the corrupted input features, *y* represents the encoded data, and *L* is a squared loss function.

In this paper, we propose a novel framework combining a sparse denoising autoencoder with the LSTM to predict anesthesia depth. Figure 4 shows the structure of SDAE-LSTM.

**Figure 5:**
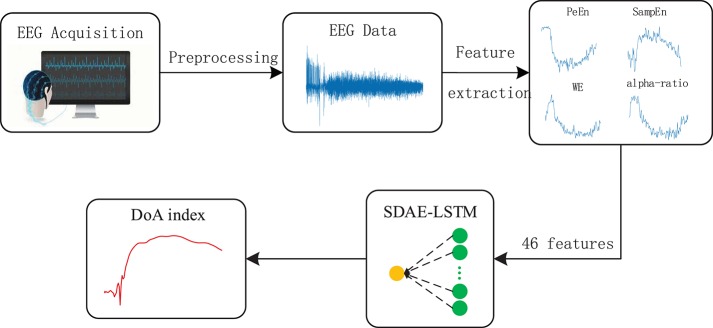
Depiction of our proposed framework. The structure of SDAE-LSTM is a contracted form, which means that 46 features are input in the SDAE-LSTM network, and there is output one index with which to monitor the DoA. The details of the SDAE-LSTM structure can be seen in Figure 4.

First, we used the notching filter to filter out power-frequency interference and the sixth-order Butterworth filter to filter out frequencies <0.8 Hz and >50 Hz. Next, the entropies, spectrum, etc., were extracted as features, and we used the wavelet threshold to smooth the features. Then, the features from the EEG data were used to train the SDAE-LSTM network, using the sevoflurane effect concentration calculated by the PK model based on end-tidal concentration as the label. SDAEs were trained one by one, after training the first SDAE; its encoder output was used as the input of the second SDAE, and the output of the second SDAE's encoder was used as the input characteristic of the LSTM for the anesthesia depth prediction training. The whole neural network was fine-tuned after the training of LSTM. The anesthesia depth index was finally obtained from the output of the SDAE-LSTM. Figure 5 illustrates the entire proposed framework.

The features we extracted represent the different patterns of EEG. Permutation entropy is robust against artifacts in EEG in the awake state (Shalbaf et al., 2013), while sample entropy can track the state of brain activity under high doses of anesthetic drugs (Shalbaf et al., 2012). Wavelet entropy can measure the degree of order/disorder of the signal and provide underlying dynamic process information associated with the signal (Rosso et al., 2001). Frequency spectrum and alpha-ratio can also be used to detect EEG activity and have been proposed as indices of anesthesia depth in previous studies (Shah et al., 1988; Drummond et al., 1991). We finally chose 46 features, including 40 frequency spectra (30–50 Hz, the spectrum every 0.5 Hz is a feature point), 2 average spectra (the average spectra 30–47 and 47–50 Hz), 3 entropies (permutation entropy, sample entropy, and wavelet entropy) and the alpha-ratio. We combined the 46 features using a 46*N feature matrix as the input to the neural network.

### 2.4. PK/PD Model

The PK/PD model describes the relationship between anesthetic drug concentration and the EEG index. It consists of two parts: pharmacodynamics and pharmacokinetics. The pharmacokinetic side of the model describes how the blood concentration of the drug changes with time, and the pharmacodynamics side represents the relationship between the drug concentration at the effect site and the measured index (McKay et al., 2006).

McKay et al. claim that the effect-site concentration of sevoflurane is related to the partial pressure of the effect site, and the partial pressure of the effect site can be calculated by the classical first-order effect site model:

$$dC_{eff}/dt=K_{eo}\left[C_{et}-C_{eff}\right]$$

where *C*_*et*_ is end-tidal concentration, *C*_*eff*_ is the effect-site concentration, and *k*_*eo*_ denotes the first-order rate constant for efflux from the effect compartment.

We used a nonlinear inhibitory sigmoid *E*_*max*_ curve to describe the relationship between *C*_*eff*_ and the measured index.

$$Effect=E_{\max}-\left(E_{\max}-E_{\min}\right)*\frac{{C_{eff}}^{\gamma }}{E{C_{50}}^{\gamma }+{C_{eff}}^{\gamma }}$$

where Effect is the EEG index, *E*_*max*_ and *E*_*min*_ are the maximum and minimum Effect respectively, *EC*_50_ describes the drug concentration that causes 50% of the maximum Effect, and γ is the slope of the concentration-response relationship.

### 2.5. Prediction Probability

To evaluate the performance of the DoA methods, the prediction probability (*P*_*k*_) statistics are used to calculate the correlation between the measured EEG index and drug effect-site concentration. The prediction probability was first proposed by Smith et al. (1996). Smith proposed using a constant to indicate the predictive performance of the anesthesia depth index.

Given two random data points *x*, *y* with different *C*_*eff*_, *P*_*k*_ describes the probability that the measured EEG index correctly predicts the *C*_*eff*_ of the two points. Let *P*_*c*_, *P*_*d*_, and *P*_*tx*_ be the respective probabilities that two data points drawn at random, independently and with replacement, from the population are a concordance, a discordance, or an *x*-only tie. The only other possibility is that the two data points are tied in observed depth *y*; therefore, the sum of *P*_*c*_, *P*_*d*_, and *P*_*tx*_ is the probability that the two data points have distinct values of observed anesthetic depth; that is, that they are not tied in *y*. *P*_*k*_ is defined as:

$$P_{k}=\frac{P_{\text{c}}+P_{tx}/2}{P_{c}+P_{d}+P_{tx}}$$

A value of 1 means that the predicted index can completely measure the depth of anesthesia, and a value of 0.5 indicates that the predicted index is completely random. Because the predictive index has a negative correlation, when the *P*_*k*_ value is < 0.5, it is replaced by 1 − *P*_*k*_.

## 3. Experimental Results

In this section, we present the experimental results of the DoA index estimation using the SDAE-LSTM network and compare it with permutation entropy, sample entropy, wavelet entropy, alpha-ratio, and our proposed network without SDAE. The sevoflurane effect concentration calculated by the PK model based on end-tidal concentration was used as the label for network training.

**Figure 6:**
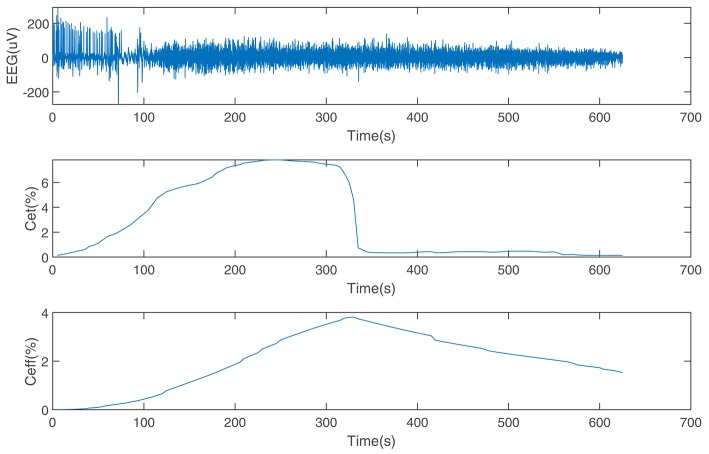
EEG data from a patient and the end-tidal concentration and effect-site concentration of sevoflurane.

The performance of the SDAE-LSTM network was tested on the dataset we introduced in section 2. This dataset consists of EEG data during anesthesia from 20 subjects, from awake to deep anesthesia. Figure 6 shows the preprocessed EEG signal, the end-tidal concentration, and the effect-site concentration of a patient during the sevoflurane induction process, from awake to anesthesia and to recovery. We input 19 training samples into the SDAE-LSTM algorithm and the regression procedure for a new test sample. The experimental result was calculated by cross-validation; each time, one subject's EEG data were selected as the test set, the order of the remaining 19 subjects' data was shuffled, and the remaining 19 subjects' data were used as the training set for SDAE-LSTM network training. The final accuracy was the average of 20 experiments. We repeated the above steps 100 times and calculated the average as the final accuracy, which shows that our method is effective and reproducible. For the baseline methods, the results were calculated by the average of the accuracy of 20 experiments. The comparison was performed on a desktop computer with an Intel Xeon CPU at 2.6 GHz and 64 GB DDR4 memory under the Windows Server 2008 OS, Matlab R2017a, and Python 3.5.

The current structure we used consists of five layers: one input layer with 46 nodes, three hidden layers with 92, 12, and 18 nodes, respectively, and one output layer with one node. The first three layers are SDAE, and the last two layers are LSTM.

**Figure 7:**
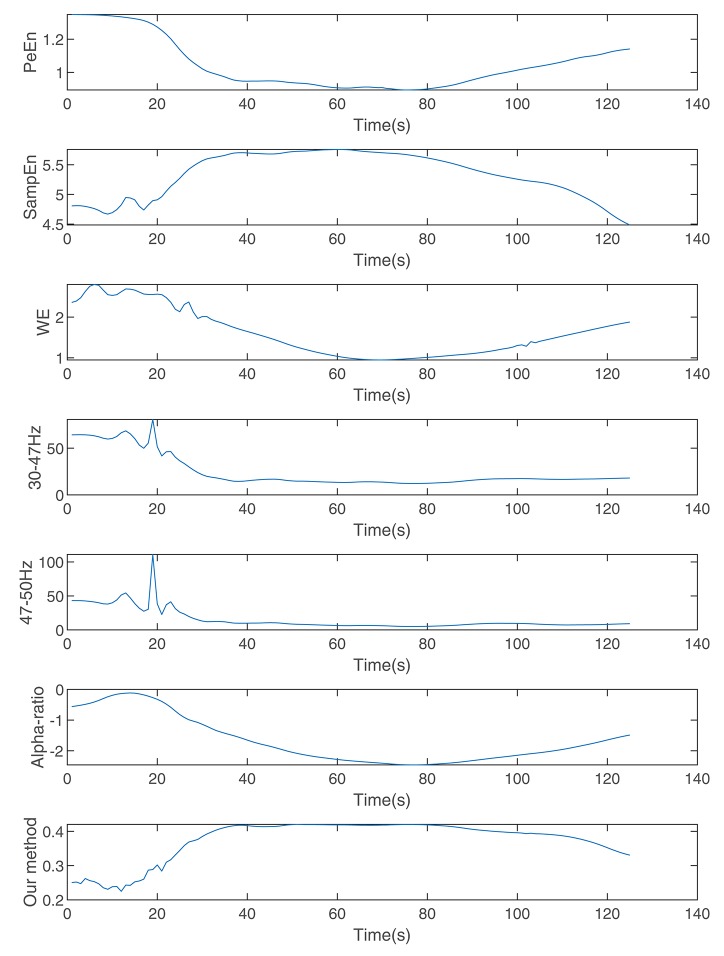
The indices calculated by wavelet entropy, sample entropy, alpha-ratio, the average spectra of 30–47 and 47–50 Hz, permutation entropy, and our method during anesthesia.

Figure 7 shows the calculated permutation entropy, sample entropy, and alpha-ratio and the index calculated during the process. It indicates that with an increase in drug effect-site concentration, the entropies decreased, and the index calculated by our method increased.

In addition, we used the Kolmogorov-Smirnov test to determine whether the *P*_*k*_ values of 20 subjects were normally distributed, and *t*-tests were used to assess whether our proposed method is more effective than other methods.

**Figure 8:**
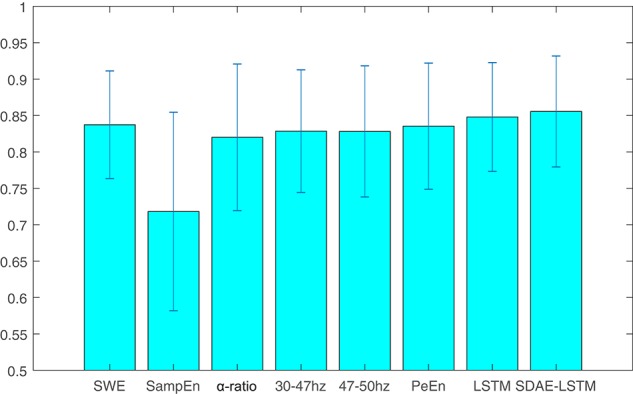
The average and standard deviation of the *P*_*k*_ values of wavelet entropy, sample entropy, alpha-ratio, the average spectra of 30–47 and 47–50 Hz, permutation entropy, LSTM, and SDAE-LSTM, respectively. The blue lines in the figure represent the variance, and the blue rectangles represent the average values.

We compare the results of our proposed network with other methods including permutation entropy, sample entropy, wavelet entropy, alpha-ratio, and the network without SDAE. The *P*_*k*_ values of these methods are presented in Table 1 and Figure 8. We can see that the *P*_*k*_ value of the permutation entropy, 0.8373, is the highest of all baseline systems and that the *P*_*k*_ value of the LSTM structure can reach 0.8479, while, using our proposed SDAE-LSTM network, the *P*_*k*_ value was highest of all methods, reaching 0.8556. Therefore, based on our results, this kind of combination has the highest *P*_*k*_ value in anesthesia monitoring. The paired *t*-tests also confirmed that the proposed method provided significantly higher accuracy than traditional methods (SDAE-LSTM>PeEn: *p* < 0: 1222, SDAE-LSTM>SampEn:*p* < 0: 00013, SDAE-LSTM>SWE: *p* < 0: 0202, SDAE-LSTM>30–47 Hz: *p* < 0: 0201, SDAE-LSTM>47–50 Hz: *p* < 0: 0431, SDAE-LSTM>alpha-ratio: *p* < 0: 0477).

**Table 1:** *P*_*k*_ values of wavelet entropy, sample entropy, alpha-ratio, the average spectra of 30–47 and 47–50 Hz, permutation entropy, LSTM, and SDAE-LSTM.

**Method**	**P_*k*_**
Wavelet entropy	0.8201 ± 0.1006
Sample entropy	0.7182 ± 0.1363
Alpha-ratio	0.8354 ± 0.0867
30–47 Hz	0.8285 ± 0.0842
47–50 Hz	0.8282 ± 0.09
Permutation entropy	0.8373 ± 0.0739
LSTM	0.8479 ± 0.0748
SDAE-LSTM	0.8556 ± 0.0762

## 4. Discussion

In this study, we propose a new method for monitoring the depth of anesthesia using EEG signals. We used wavelet entropy, permutation entropy, sample entropy, alpha-ratio, and frequency spectrum to extract features from EEG signals. However, EEG is a complex dynamic signal with multiple linear and nonlinear features and it is very sensitive to noise. Using only one linear method or nonlinear method cannot analyze all aspects of brain activity. These traditional methods may vary from patient to patient and type of surgery. Moreover, muscle relaxants, anesthetics, and other similar drugs used during surgery affect the EEG, thus making the analysis of clinical symptoms unreliable (Shalbaf et al., 2015).

Therefore, a new method based on LSTM and an SDAE is proposed. LSTM is a neural network for processing sequence data. Compared with the general neural network, it is more suitable for processing and predicting important events with relatively long intervals and delays in the time series. Therefore, it can analyze the temporal information present in EEG. An SDAE is an improved unsupervised deep neural network. The sparseness constraint applied in the hidden layer of the network makes the representation of data sparse. Applying the denoising autoencoder to destroy the input data helps enhance the robustness of the system, making it suitable for the analysis of EEG signals that have a lower signal-to-noise ratio.

Pharmacokinetic/Pharmacodynamic (PK/PD) modeling and prediction probability were used to evaluate the effectiveness of the SDAE-LSTM model for monitoring DoA. PK/PD modeling can be sued to establish the relationship between the concentration of the anesthetic at the effect site and the EEG index. This method has been used to assess the proposed EEG index successfully (McKay et al., 2006). The PK side we used describes the changes in drug concentration in blood over time. The prediction probability was first proposed by Smith et al. (1996), proposing a constant to express the predictive accuracy of DoA (Smith et al., 1996).

We used a dataset collected by a New Zealand hospital containing 20 sets of patient data from the use of sevoflurane anesthesia methods, which recorded the entire process from the beginning to the end of anesthesia. Cross-validation was used to assess whether our method is effective. We compared the proposed method with the traditional methods. The results show that our method can achieve a *P*_*k*_ value of 0.8556 for predicting the depth of anesthesia, which is about 2% higher than the baseline methods and is increased by 0.77% compared with using a LSTM network only.

## 5. Conclusion

This paper provides a method for monitoring the DoA using EEG, which helps to provide a safer, reliable, and effective clinical environment for anesthetized patients. In this study, permutation entropy, wavelet entropy, sample entropy, alpha-ratio, and frequency spectrum are used to extract the features of EEG signals. These extracted features are applied to an SDAE-LSTM network. The method was compared with the traditional methods and achieved good results. Therefore, this method of combining the hybrid features in an SDAE-LSTM network can be used in future studies on anesthesia depth monitoring.

## Data Availability Statement

The dataset for this article is not publicly available. We asked for the dataset from one of the authors of McKay et al. (2006) by email. Requests to access the datasets should be directed to logan.voss@waikatodhb.health.nz.

## Author Contributions

QiaW and JL contributed conception and design of the study. RL and CL achieved the algorithm and finished numerical experiment. RL wrote the draft of the manuscript. QiW preprocessed the EEG data. QZ performed the statistical analysis. All authors contributed to manuscript revision, read, and approved the submitted version.

### Conflict of Interest

The authors declare that the research was conducted in the absence of any commercial or financial relationships that could be construed as a potential conflict of interest.
